# Remediation of Cr(VI)-Contaminated Soil by Nano-Zero-Valent Iron in Combination with Biochar or Humic Acid and the Consequences for Plant Performance

**DOI:** 10.3390/toxics8020026

**Published:** 2020-04-03

**Authors:** Yuhuan Sun, Fangyuan Zheng, Wenjie Wang, Shuwu Zhang, Fayuan Wang

**Affiliations:** 1 College of Environment and Safety Engineering, Qingdao University of Science and Technology, Qingdao 266042, China; yhsun@qust.edu.cn (Y.S.); zhengfy9890@163.com (F.Z.); wwj09301230@163.com (W.W.); zhangshuwu@126.com (S.Z.); 2 Key Laboratory of Soil Resources and Environment in Qianbei of Guizhou Province, Zunyi Normal University, Zunyi 563002, China

**Keywords:** zero-valent iron, phytotoxicity, chromium-contaminated soil, biochar, humic acid

## Abstract

Nano-scale zero-valent iron (nZVI) is among the most common nanoparticles widely used for the treatment of various environmental contaminants. However, little is known about the combined effects of nano-zero-valent iron (nZVI) and other soil amendments on soil remediation and plant performance. For the first time, we studied the remediation of Cr(VI)-contaminated soil using bare nZVI (B-nZVI) and starch-supported nZVI (S-nZVI) in combination with either biochar (BC) or humic acid (HA), and the consequent effects on plant growth and Cr accumulation. Both S-nZVI and B-nZVI decreased the contents of Cr(VI) and available Cr in soil, but increased available Fe content, with S-nZVI generally showing more pronounced effects at a higher dose (1000 mg/kg). B-nZVI exerted no inhibition and even stimulation on plant growth, but 1000 mg/kg S-nZVI produced significant phytotoxicity, resulting in decreased plant growth, low chlorophyll content in leaves, and excessive accumulation of Fe in roots. Each nZVI decreased shoot and root Cr concentrations. BC and HA produced synergistic effects with nZVI on Cr(VI) removal from soil, but HA decreased soil pH and increased the availability of Cr and Fe, implying a potential environmental risk. Addition of BC or HA did not alter the effects of either nZVI on plant growth. In conclusion, combined application of 100 mg/kg nZVI and BC could be an ideal strategy for the remediation of soil contaminated with Cr(VI), whereas high-dose S-nZVI and HA are not recommended in the remediation of agricultural soils for crop production or in the phytostabilization of Cr(VI).

## 1. Introduction

Chromium (Cr) can enter the environment via a variety of human activities, including mining, smelting, and electroplating, leading to potential contamination to soil, surface water, or groundwater [1,2,3]. Among several redox states of Cr, Cr(III) and Cr(VI) represent the most stable and common forms. Generally, oxidative Cr(VI) has higher toxicity to organisms than reductive Cr(III). Cr(VI) has been classified as a class I carcinogen to humans by the International Agency for Research on Cancer (IARC) and is listed as one of the priority contaminants [4]. Cr(VI) may migrate easily in soil and accumulate in plants (especially crops), posing a health risk to human and animals [5]. For this reason, various technologies have been developed for the remediation of Cr(VI)-contaminated sites [6,7]. Among them, chemical and microbial remediation techniques, particularly the reduction of Cr(VI) to the less poisonous Cr(III), represent the most interesting strategies [2,8].

Recently, nanoremediation based on nano zero-valent iron (nZVI) has attracted increasing attention due to its environmental friendliness, high efficiency, and wide applications [9]. nZVI has been used to reduce leaching of toxic metals through adsorption, complexation and chemical reduction processes [10,11,12,13,14,15,16]. Numerous studies have reported applications of nZVI in the remediation of soils contaminated with Cr [17,18,19,20] as well as other toxic metals [14,15,16] and organic contaminants [21]. However, bare nZVI readily becomes aggregated and oxidized in aerobic environments, leading to a low remediation efficiency. Meanwhile, nZVI (especially in excess) will inevitably induce adverse ecological effects during the remediation process [22,23,24,25]. For instance, nZVI generally exhibits “low-dose stimulation and high-dose inhibition” effects on plant growth via multiple mechanisms [22]. Combined applications of physical, chemical and biological methods have been recommended to enhance the performance of nZVI and reduce its possible disadvantages [21].

Biochar (BC) and humic acid (HA) are both among amendments that are widely used in soil remediation. As a type of carbon-rich material with rich porous structures, various functional groups, a high pH and a high cation exchange capacity, BC generally possesses a high adsorption capacity that potentially contributes to the immobilization of heavy metals [26,27], including Cr [28,29]. Previously, Cr(VI) reduction in soil was enhanced by modified manure-derived BCs, and the toxicity of Cr(VI) was alleviated via adsorption, immobilization and reduction [28]. HA contains heterogeneous organic molecules with abundant functional groups, and can enhance [30] or decrease [31] the mobility of metals in soil, thus contributing to soil washing or soil immobilization. Furthermore, HA represents a significant reservoir of electron donors and thus participates in reduction of Cr(VI) [32]. However, in another study, HA did not promote reduction of Cr(VI) to Cr(III), but led to the formation of Cr(VI)–HA micelles via supramolecular chemical processes [33]. Recently, organic acids were found to exert synergistic effects with BC on Cr(VI) reduction [34,35]. Notwithstanding, whether BC and HA can be used to assist nZVI-remediation of Cr(VI) still remains unclear. 

Considering the above context, combined use of nZVI with soil amendments such as BC and HA may produce better remediation effects for Cr(VI)-contaminated soil. We hypothesize that combined application of BC or HA may enhance nZVI’s performance in Cr(VI) remediation, and decrease the possible phytotoxicity induced by nZVI. Here, using bare-nZVI and starch-stabilized nZVI as targets, we conducted a pot culture experiment to assess the effects of nZVI in combination with BC or HA on Cr(VI) removal from soil and the growth and accumulation of Cr in mung bean. The results may provide evidence for combined applications of nZVI with other amendments and phytoremediation approaches. 

## 2. Materials and Methods

### 2.1. Soil Sample Preparation

Cr-free and Cr-contaminated soils were selected for this study. The Cr-free soil was collected from a local farmland in Qingdao, China (36°26′53.89′′ N, 120°08′55.22′′ E), and the Cr-contaminated soil was sampled from the Cr-containing slag heap site of Qingdao Red Star Chemical Plant (36°12′21′′ N, 12°23′18′′ E). The detailed characteristics of the two kinds of soil samples were described in our previous studies [7,36]. The soils were first air-dried, then sieved (2 mm). Background values of Cr(VI) and total Cr in the soils were determined. In order to obtain Cr-contaminated soil with an environmentally realistic concentration of 50 mg/kg, the two soils were mixed in a certain proportion and aged for one week at room temperature after soil preparation was completed. Soil properties were determined prior to pot culture (Table 1).

### 2.2. Synthesis and Characterization of Starch-Stabilized nZVI

Due to advantages such as low-cost and biodegradability, starch has previously been used for surface-modification, in order to minimize aggregation and enhance the stability and reactivity of nZVI [37]. Starch-stabilized nZVI (S-nZVI) was synthesized using the borohydride reduction method, as described in our previous study [38], with average particle size of 69.5 nm, a zeta potential of −14.2 mV and an average surface area of 46.3 m^2^/g (Figure S1).

### 2.3. Characterization of Bare nZVI

Bare nZVI (B-nZVI) was purchased from Beijing Deke Daojin Science and Technology Co., Ltd. (Beijing, China), with following properties: purity 99.9%, average particle size 30–50 nm and specific surface area 40–60 m^2^/g. TEM imaging and XRD pattern of B-nZVI are provided in Figure S2. 

### 2.4. Biochar and Humic Acid

Pine shoots-derived biochar (BC) was obtained from Qingdao Biochar Environmental Biological Engineering Co., Ltd., Qingdao, China, with the following properties: pH 9.64, average specific surface area 139.4 m^2^/g, C 86.15%, H 3.17%, N 0.29%, S 0.51%, O 7.36%, Cr 32.45 ng/g, zeta potential −26 mV and average particle size 9469.6 nm. SEM images and FTIR spectra of the BC are shown in Figure S3. 

Humic acid (HA) was obtained from Tianjin Guangfu Fine Chemical Research Institute, Tianjin, China. The purity was >99% and the pH was 5.92. The HA contained ≥90% fulvic acid, 10% ash, 8% water, 0.3% Fe and <1% insoluble matter. 

### 2.5. Pot Culture Experiment

We designed a trifactorial experiment, including (1) two types of nZVI (i.e., B-nZVI and S-nZVI); (2) three nZVI doses (i.e., 0, 100 and 1000 mg/kg); and (3) two doses of BC or HA (i.e., 0% and 1% (*w/w*)). Each treatment had four replicates. An appropriate amount of nZVI was mixed thoroughly into soil to achieve the target doses. Next, 1% (*w/w*) of BC or HA was mixed into the soil samples. Seeds of mung bean (*Vigna radiata*) from a local market in Shangqiu in Henan Province, China were used for the pot culture experiment. The experiment was conducted according to the procedure described previously [39]. Twenty surface-sterilized seeds were placed in each pot (180 mm diameter and 110 mm height) containing 800 g soil. The seedlings were thinned and 10 were kept evenly in each pot. The pots were randomly arranged in a growth chamber with a day/night (12/12 h) temperature regime of 25–28/20–23 °C, a light intensity of 10000 Lux and relative humidity of 50–55%. Deionized water was irrigated every other day to maintain a consistent soil moisture of 50–60%. 

### 2.6. Plant Harvesting

The mung bean plants were harvested 30 days after seed sowing. Fresh weights (FWs) of the shoots and roots were measured respectively after cleaning. The dry weights (DWs) were determined after oven-drying the fresh materials at 70 °C for 24 h. 

### 2.7. Plant and Soil Analysis

On the date of harvesting, fresh leaves were sampled for measurement of chlorophyll content. The contents of chlorophyll a and b were determined using an ultraviolet spectrophotometer (UV spectrophotometer, UV-1800, Shimadzu, Kyoto, Japan) after extracting the pigments from the leaves in a 95% ethanol solution [40]. Total chlorophyll content was defined as the summation of chlorophyll a and b.

The dried shoot (0.5 g) and root (0.2 g) samples were ground and then digested using HNO_3_. Cr concentration in the solution was estimated with an inductively coupled plasma mass spectrometer (ICP-MS, iCAP RQ, Thermo Fisher Scientific Inc., Waltham, MA, USA). Fe concentration in the solution was determined with an atomic absorption spectrophotometer (FAAS, AA-7000, Shimadzu, Kyoto, Japan).

To observe the phytotoxic symptoms and iron element distribution at the cell level, the microscopic structures were observed according to the detailed procedure described in [41]. Briefly, fresh root samples were cut with a ultramicrotome (EM UC7, Leica, Nussloch, Germany) then characterized by transmission electron microscopy (TEM, FEI, Tecnai G2 Spirit, Hillsboro, OR, USA) coupled with energy-dispersive X-ray spectroscopy (EDS, EDAX, Apollo XLT SDD, Philadelphia, PA, USA).

The post-harvest soil from each pot was mixed thoroughly and 50-g soil samples were taken for analysis of soil properties. Available Fe and Cr concentrations were determined using FAAS after extracting using DTPA solution (0.005 M DTPA, 0.1 M triethanolamine, 0.01 M CaCl_2_, pH 7.3) [42]. The content of Cr(VI) in soil was determined using the 1,5-diphenylcarbazide spectrophotometric method (GB/T 15555.4-1995). Soil pH (soil–water ratio, 1:2.5, *w*/*v*) was determined using a pH meter (pHS-3C, Sanxin, Shanghai, China).

### 2.8. Data Analyses

One-way analysis of variance and multi-way analysis of variance were conducted with a 5% probability level using SPSS 23.0 (IBM SPSS, Chicago, IL, USA). Significance among treatments was compared using a Duncan’s multiple range test at *p* < 0.05. All figures were generated using OriginPro9 (OriginLab, Northampton, MA, USA), and the results are presented as means ± standard deviation (SD). 

## 3. Results

### 3.1. Cr(VI) Concentrations in Soil

**Figure 1:**
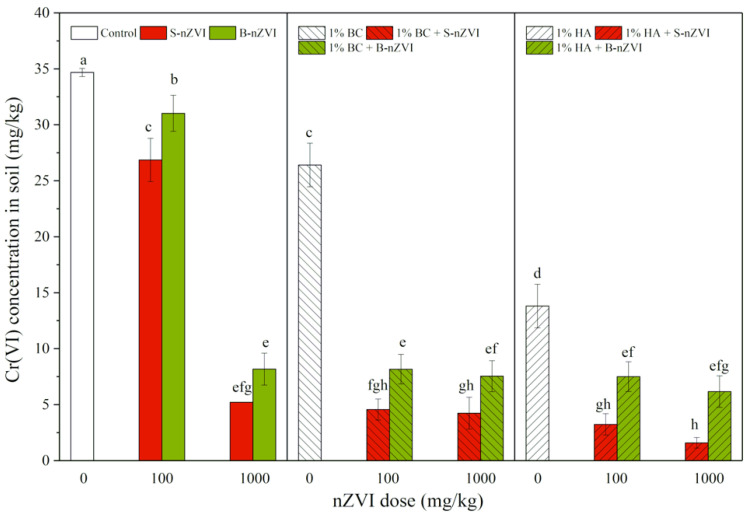
Cr(VI) concentrations (means ± SD, *n* = 4) in soil with nZVI alone or in combination with biochar (BC) or humic acid (HA). Different letters on the bars indicates significant differences among all means in different treatments using a one-way ANOVA followed by a Duncan’s multiple range test (*p* < 0.05). Three-way ANOVA results are shown in Table 2.

As shown in Figure 1, both S-nZVI and B-nZVI decreased Cr(VI) concentrations in soil, with more pronounced effects in S-nZVI, especially at a higher dose (1000 mg/kg). A single application of HA had higher decreasing effects than BC alone. Combined applications of nZVI and BC or HA greatly decreased Cr(VI) concentrations in soil, compared to a single application. The most significant decrease in Cr(VI) concentrations was observed in a combination treatment with 1000 mg/kg S-nZVI and HA, with 95% of Cr(VI) removed as compared to the control.

Three-way ANOVA results revealed that nZVI type, dosage, use of BC or HA, and type of nZVI interaction (nZVI-BC or nZVI-HA) all had significant impacts on soil Cr(VI) concentrations, with better effects of S-nZVI than B-nZVI, as well as synergistic interactions between nZVI and both BC and HA (Table 2). 

### 3.2. DTPA-Extractable Cr and Fe Concentrations in Soil

**Figure 2:**
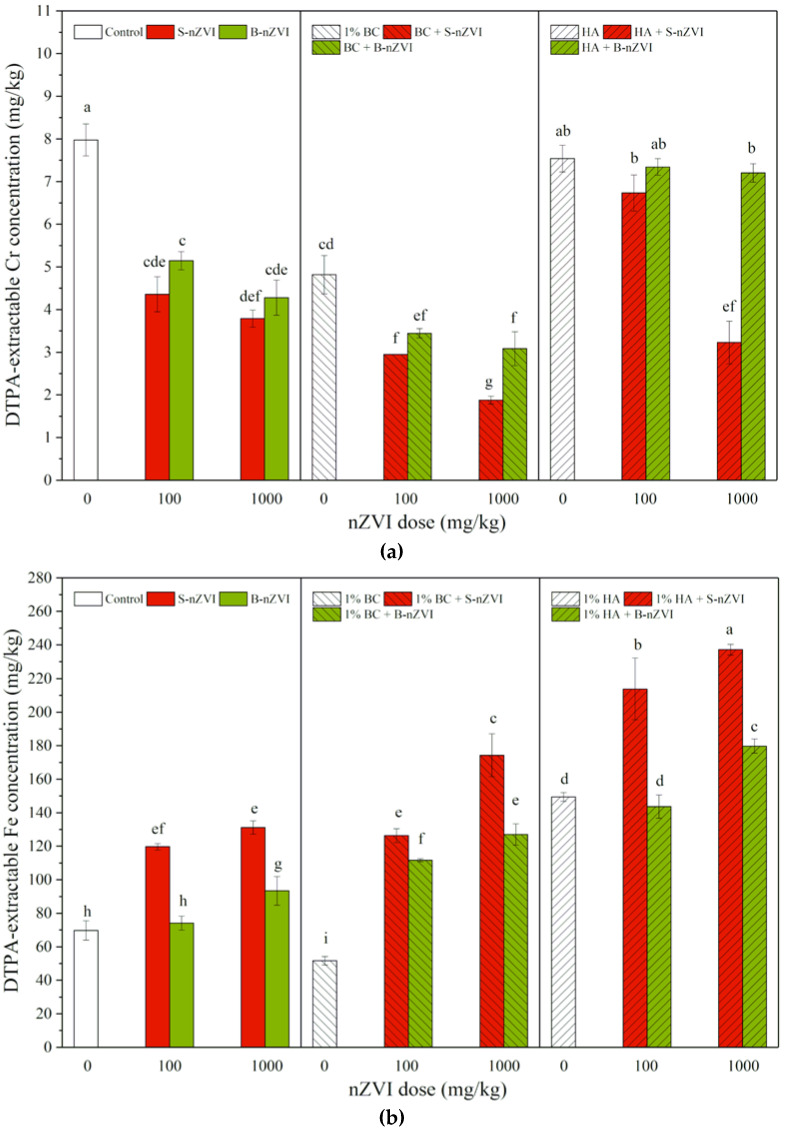
DTPA-extractable Cr (**a**) and Fe (**b**) concentrations (means ± SD, *n* = 4) in soil with nZVI alone or in combination with BC or HA. Different letters on the bars indicate significant differences among all means in different treatments using a one-way ANOVA followed by a Duncan’s multiple range test (*p* < 0.05). Three-way ANOVA results are shown in Table 2.

In comparison with the control, B-nZVI and S-nZVI both significantly decreased DTPA-extractable Cr concentrations in soil, but there were no significant differences between two nZVI types and two doses (Figure 2a). Addition of BC alone or in combination with nZVI decreased DTPA-extractable Cr concentrations in soil, and the combinations produced more significant effects. HA alone showed no significant effects, but decreased DTPA-extractable Cr concentrations in soil when applied in combination with nZVI (except for HA + 100 mg/kg S-nZVI). Compared to nZVI alone, a combined application with BC showed a decreasing trend in DTPA-extractable Cr, but a combined application with HA exhibited an increasing trend. Different from one-way ANOVA results, three-way ANOVA results showed that soil DTPA-extractable Cr was significantly influenced by the variables separately, including the interaction between nZVI type and dosage and the interactions among nZVI type, dosage and HA (Table 2).

Compared to the control, exposure to B-nZVI and S-nZVI (except 100 mg/kg B-nZVI alone) significantly increased DTPA-extractable Fe concentrations in soil, particularly at higher doses, but S-nZVI always had a stronger promoting effect than B-nZVI (Figure 4b). Addition of BC alone decreased DTPA-extractable Fe concentrations in soil, but HA increased them. Compared to nZVI alone, combined treatments with HA or BC had higher DTPA-extractable Fe concentrations in soil, with HA producing more promoting effects than BC. Three-way ANOVA results showed that HA alone (*F* = 1237.1) mostly influenced DTPA-extractable Fe, and significantly interacted with nZVI type and dosage (Table 2).

### 3.3. Soil pH

**Figure 3:**
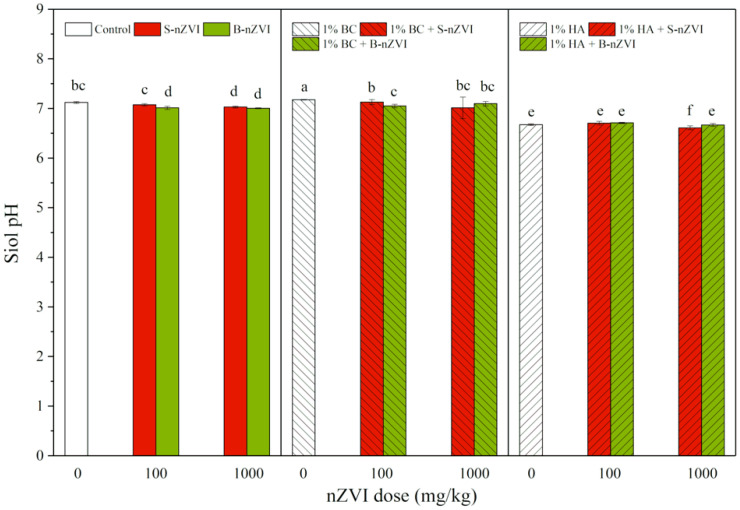
pH value (means ± SD, *n* = 4) of soil with nZVI alone or in combination with BC or HA. Different letters on the bars indicate significant differences among all means in different treatments using a one-way ANOVA followed by a Duncan’s multiple range test (*p* < 0.05). Three-way ANOVA results are shown in Table 2.

Compared to the control, addition of B-nZVI and S-nZVI generally decreased soil pH, whereas BC increased and HA decreased soil pH, with the most significant decrease in combination treatment with 1000 mg/kg S-nZVI and HA (Figure 3). Three-way ANOVA results showed that HA produced the most significant impacts on soil pH, followed by BC (Table 2). 

### 3.4. Plant Biomass

**Figure 4:**
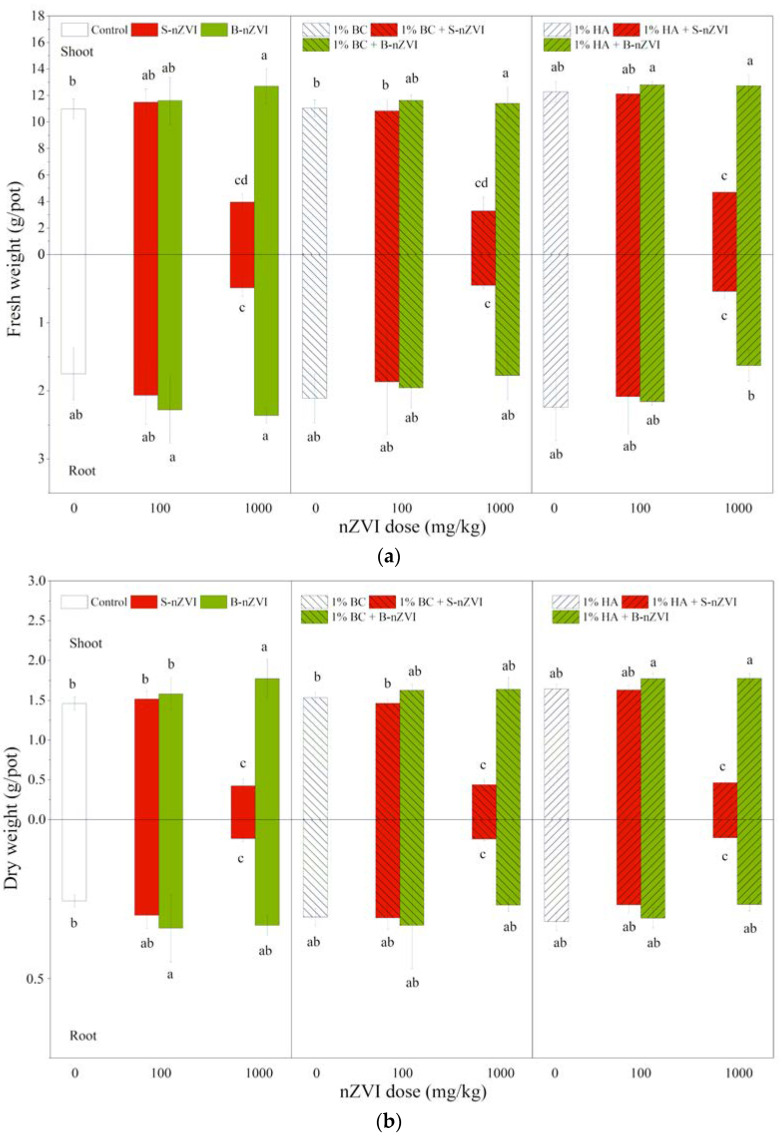
Shoot (above X-axis) and root (below X-axis) fresh (**a**) and dry (**b**) weights (means ± SD, *n* = 4) of mung bean seedlings exposed to nZVI alone or in combination with BC or HA. Different letters on the bars indicate significant differences among all means in different treatments using a one-way ANOVA followed by a Duncan’s multiple range test (*p* < 0.05). Three-way ANOVA results are shown in Table 2.

B-nZVI and S-nZVI had different impacts on plant biomass (Figure 4). Compared to the control, 100 and 1000 mg/kg B-nZVI did not inhibit and in some cases even enhanced plant biomass. For example, the seedlings exposed to 1000 mg/kg B-nZVI had higher shoot fresh and dry weights. However, the effects of S-nZVI highly varied with dose. S-nZVI showed no significant effects at 100 mg/kg, but the seedlings exposed to 1000 mg/kg S-nZVI exhibited poisoning symptoms, with yellow leaf surface four days after germination, and black spots on leaves eight days later. Thereafter, the symptoms in leaves became more severe, and began to fall off after exposure to S-nZVI for 16 days. 

Compared to the control seedlings and those treated with nZVI, BC and HA did not show marked effects on the fresh or dry weights of shoots or roots when applied alone or in combination with nZVI (Figure 4). For example, the seedlings receiving 1000 mg/kg S-nZVI always had the lowest fresh and dry weights, irrespective of BC or HA. 

Three-way ANOVA results showed that nZVI type and dosage and their interactions had significant effects on plant fresh and dry weights (Table 2), but HA interacted with nZVI type and dosage, which effected root growth.

### 3.5. Chlorophyll Content

**Figure 5:**
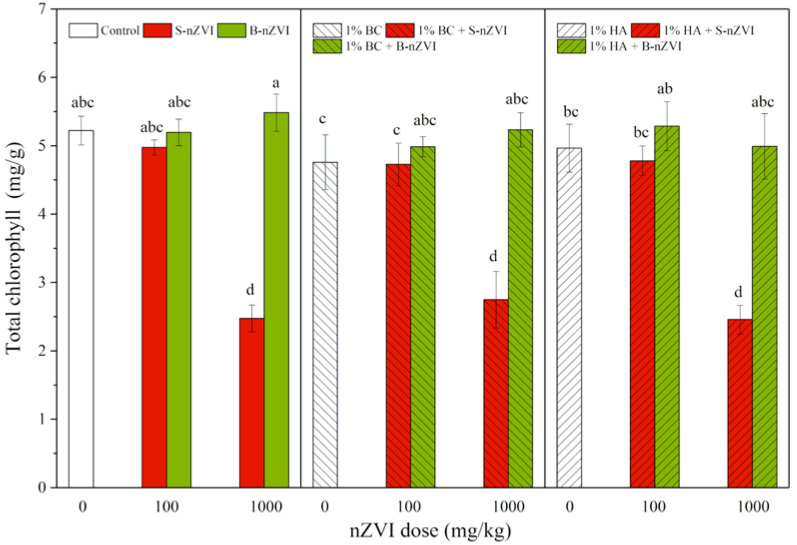
Total chlorophyll (means ± SD, *n* = 4) in leaves of mung bean seedlings exposed to nZVI alone or in combination with BC or HA. Different letters on the bars indicate significant differences among all means in different treatments using a one-way ANOVA followed by a Duncan’s multiple range test (*p* < 0.05). Three-way ANOVA results are shown in Table 2.

As shown in Figure 5, the seedlings exposed to 1000 mg/kg S-nZVI always had the lowest total chlorophyll content in leaves. Combined applications with BC or HA did not further change the trend. All other treatments did not significantly influence the chlorophyll content in leaves, and no significant difference was observed among them. Three-way ANOVA results showed that nZVI type and dosage had marked impacts on chlorophyll content (Table 2).

### 3.6. Cr and Fe Concentrations in Plants

**Figure 6:**
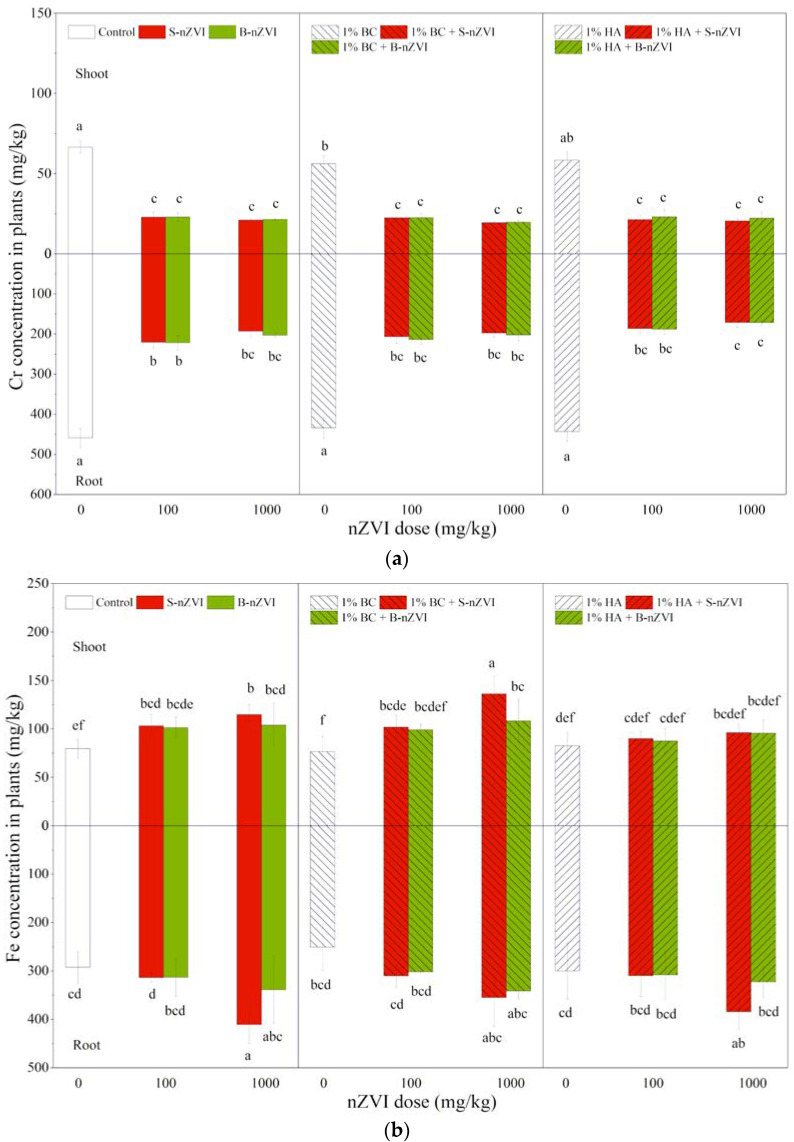
Cr (**a**) and Fe (**b**) concentrations (means ± SD, *n* = 4) in shoots (above *X*-axis) and roots (below *X*-axis) of mung bean seedlings exposed to nZVI alone or in combination with BC or HA. Different letters on the bars indicate significant differences among all means in different treatments using a one-way ANOVA followed by a Duncan’s multiple range test (*p* < 0.05). Three-way ANOVA results are shown in Table 2.

As shown in Figure 6a, both B-nZVI and S-nZVI significantly decreased shoot and root Cr concentrations, with a decrease of about 50%, but there were no significant differences between the two nZVI types and the two doses. Compared to the control, addition of BC alone decreased shoot Cr concentration, while HA alone showed significant impacts on root Cr concentrations (Table 2). Combined applications with BC or HA did not further influence nZVI’s effects on Cr concentrations in shoots or roots.

In most cases, S-nZVI and B-nZVI alone or in combination with BC increased shoot Fe concentrations, but the significant effects were only observed for root Fe concentrations at 1000 mg/kg S-nZVI (Figure 6b). HA did not influence shoot and root Fe concentrations when applied alone, and did not change either nZVI’s impacts. Three-way ANOVA results showed that plant Fe accumulation was only significantly influenced by nZVI dosage (Table 2). 

## 4. Discussion

The most important finding of our present study is that B-nZVI at both doses did not inhibit, and even promoted, mung bean growth, where S-nZVI at 1000 mg/kg was highly phytotoxic. Understandably, the difference can be attributed to the starch stabilization. B-nZVI easily aggregates into larger particles or becomes oxidized under aerobic conditions, which can explain its lower toxicity and occasional beneficial effects. However, starch stabilization enhances the dispersity and reactivity of S-nZVI, which consequently increases its tendency to be adhered and penetrated into roots. Accompanied by excess accumualtion of total Fe (Figure 6) and nZVI (Figure S4) in roots, severe imparirments such as plasmolysis, contraction of cytoplasm and sometimes cytomembrane breakage were observed in root cells exposed to S-nZVI (Figure S4). This could confirm that toxicity of S-nZVI indeed ocurred within root cells, a finding that is in agreement with our previous hydroponic culture expeiment [38]: S-nZVI at 600 mg/L produced signifciant phytotoxicity in mung bean, such as an altered nutritional status, excess Fe in roots (>400 mg/kg), formation of a coating on root surfaces and penetration of nZVI into roots. Our present finding confirms that, when applied in soil remediation, bare nZVI is generally friendly for plant growth, while high doses of S-nZVI can cause phytotoxicty. This should be taken into account for its application in the remediation of agricultural soils for crop production, or a combined application for phytoremediation.

Numerous studies have shown that nZVI can be used in the remediation of soils contamianted with Cr [17,18,19,20]. Our present study further confirms that both B-nZVI and S-nZVI greatly decreased Cr(VI) concentrations in soil, but that S-nZVI was overall more efective than B-nZVI, particularly at a higher dose (three-way ANOVA results, see Table 2). This proves that starch stabilization indeed helps to maintain the dispersity of nanoparticles and/or the reactivity of S-nZVI as a reductant. Furthermore, as an organic substance, the starch in S-nZVI may function as a reducing agent and directly react with Cr(VI), ultimately contributing to Cr(VI) removal. However, considering its phytoxicity, high doses of S-nZVI may cause higher environmental risk than B-nZVI. An appropriate dose should be carefully selected for safe environmental applications.

More interestingly, both BC and HA were effective in the removal of Cr(VI) from soil, both alone or in comibation with nZVI (Figure 1). However, their remediation mechanisms are probably different. BC can immobilize heavy metals via direct mechanisms such as electrostatic attraction, ion exchange, complexation and precipitation, as well as indirect changes in soil properties, and thus decrease the mobility/bioavailability of metals in contaminated soils and thus their uptake by plants [26,43]. Therefore, as a support material to stabilize nZVI, BC can enhance the adsorption removal of Pb^2+^, Cu^2+^, Zn^2+^ [44] and Cr(VI) [10], as well as the in situ remediation of Cr(VI) in soil, while reducing the phytotoxicity of Cr and leachable Fe [18,45]. In our present study, BC increased soil pH. and decreased Cr(VI) and available Cr concentrations in soil; however, it enhanced available Fe concentrations in soil when coexisting with nZVI, and did not alleviate S-nZVI phytotoxicity (Figure 4). The possible reasons for this may be: (1) the presence of BC may reduce or delay the oxidation and corrosion of nZVI; and (2) the adsorption of Fe^2+^ and Fe^3+^ onto BC can prevent their precipitation. This also accounts for the phytotoxicity of S-nZVI, which was mainly from the nZVI and the leached Fe, but not related with Cr(VI) in the soil. 

As is a type of soluble organic molecule with low pH, HA generally enhances the bioavailability and mobility of heavy metals in soil [30,46,47]. As expected, HA decreased soil pH, but resulted in higher available Cr and Fe concentrations in soil when applied in combination, as opposed to the nZVI alone (Figure 2), indicating that the HA-induced decrease in Cr(VI) was mainly from reduction of Cr(VI) to Cr(III) [32]. Subsequently, HA may solubilize the Cr(III) through complexation [48], leading to the formation of a soluble Cr(III)-complex. In another study, the presence of HA increased the concentration of dissolved Fe due to the formation of soluble Fe-humate complexes [49]. Hence, unlike BC, HA may promote the mobility of Cr and Fe (Figure 2), thus increasing their environmental risks. Overall, both BC and HA can be used in the nZVI remediation of Cr(VI)-contaminated soil, but BC is more environmentally friendly than HA. 

Soil pH is a key factor determinging the mobility of heavy metals and their uptake by plants [50,51], as well as environmental transformations of nZVI [22]. The corrosion of nZVI in soil generally increases soil pH [18,52], but sometimes corrosion decreases [53] or exerts no significant impact on soil [54], depending on the nZVI type and dose, contaminants and soil properties. Our present study found that soil pH was slightly decreased by both nZVI. The possible reasons can be attributed to the high cation exchange capacity (CEC) and organic matter content of the soil we used. Comparatively, soil pH increased with BC, but greatly decreased with HA (Figure 3 and Table 2), which was apparently due to the different pH values of these two materials. A lower soil pH is more favourable for Cr(VI) reduction by nZVI [54,55], which helps to explain why HA had a higher Cr(VI) removal than BC when they were jointly applied with nZVI. 

In addition to decreased biomass, we also observed much lower chlorophyll contents in leaves of mung bean seedlings exposed to 1000 mg/kg S-nZVI (Figure 5). Fe participates in various physiological processes within higher plants, such as photosynthesis, respiration, and chlorophyll biosynthesis [56], and a previous study found that the addition of nZVI in soil increased the total Fe content in plant tissues, while greatly inhibiting the transport of active Fe from root to shoot and thus lowering active Fe content in the shoot, ultimately leading to iron-deficient chlorosis in rice [42]. In our present study, although all the seedlings had a normal range of Fe content in shoots (60–300 mg/kg) [57], the active Fe in leaves of S-nZVI-exposed seedlings may have been deficient, hindering chlorophyll biosynthesis and photosynthesis in the process. Furthermore, the high dose of S-nZVI caused serious damage in roots, which may further disturb plant nutrient uptake and balance. For example, S-nZVI was found to decrease Mn content in mung bean shoots and cause the appearance of black spots in leaves [38]. In conclusion, S-nZVI may induce nutrient deficiency and interfere with the biosynthesis and subsequent photosynthesis of chlorophyll, resulting in plant growth reduction. 

## 5. Conclusions

To our knowledge, this is the first study evaluating nZVI remediation of Cr(VI)-contaminated soil assisted by BC or HA and the consequent phytotoxicity. Overall, both S-nZVI and B-nZVI were effective in Cr(VI) removal from soil, although the effects were more pronounced for S-nZVI at a higher dose. Combined applications with BC or HA further enhanced the remediation effects of both nZVI, but HA increased the availability of Fe and Cr in soil. Both types of nZVI decreased Cr accumulation in mung bean seedlings, but only S-nZVI displayed “high-dose inhibition effects” on plant growth, suggesting that B-nZVI is more environmentally friendly for plants than S-nZVI when applied at a high dose. Taking plant performance, Cr(VI) removal and Fe release altogether, 100 mg/kg of B-nZVI or S-nZVI in combination with BC is determined to be an ideal remediation strategy. Given that high-dose S-nZVI can cause phytotoxicity, and HA promotes the increased release of available Fe and Cr, hence S-nZVI and HA are not recommended in the remediation of agricultural soils for crop production and phytostabilization of Cr(VI).

## Figures and Tables

**Table 1:** Physicochemical properties of the soil used for pot culture.

Soil Parameters	Value
pH	6.78
Cr(VI) (mg/kg)	50.0
Total Cr (mg/kg)	928.2
Total Cd (mg/kg)	0.15
DTPA-extractable Fe (mg/kg)	62.8
DTPA-extractable Zn (mg/kg)	0.96
Cation exchange capacity (CEC) (cmol/kg)	7.22
Organic matter (g/kg)	12.6
Total N (g/kg)	0.78
Available P (mg/kg)	289.4
Available K (mg/kg)	170.6
Nitrate N (mg/kg)	19.6
Sand (%)	68.3
Silt (%)	22.7
Clay (%)	9.0

**Table 2:** Significance levels (*F* values) of nZVI type and dosage, BC or HA addition level and their interactions on measured variables in a three-way ANOVA analysis.

Variables	nZVI Type	nZVI Dosage	BC	HA	nZVI Type × Dosage	nZVI Type × BC	nZVI Dosage × BC	nZVI Type × Dosage × BC	nZVI Type × HA	nZVI Dosage × HA	nZVI Type × Dosage × HA
Soil Cr(VI)	45.2 ***	83.0 ***	294.3 ***	615.3 ***	0.0 ns	0.0 ns	257.9 ***	0.1 ns	0.4 ns	234.5 ***	0.3 ns
DTPA-Cr	118.7 ***	82.9 ***	202.2 ***	58.5 ***	38.2 ***	0.5 ns	0.0 ns	2.5 ns	30.8 ***	13.7 **	37.8 ***
DTPA-Fe	429.5 ***	153.2 ***	40.0 ***	1237.1 ***	2.0 ns	3.9 ns	9.0 **	13.8 **	16.5 ***	7.0 *	0.2 ns
Soil pH	1.1 ns	11.7 **	46.9 ***	1511.8 ***	5.7 *	0.1 ns	1.5 ns	0.0 ns	11.3 **	4.4 *	0.1 ns
Shoot FWs	250.1 ***	174.2 ***	1.5 ns	8.5 **	184.1 ***	0.1 ns	0.4 ns	1.9 ns	0.0 ns	0.7 ns	1.0 ns
Root FWs	34.0 ***	71.5 ***	0.4 ns	0.0 ns	40.9 ***	0.6 ns	0.2 ns	1.9 ns	0.8 ns	1.0 ns	4.7 *
Shoot DWs	109.4 ***	83.9 ***	0.4 ns	3.0 ns	68.2 ***	1.0 ns	1.3 ns	2.9 ns	0.0 ns	0.8 ns	0.1 ns
Root DWs	54.9 ***	85.7 ***	0.1 ns	0.0 ns	55.9 ***	0.5 ns	0.7 ns	1.3 ns	0.1 ns	0.0 ns	4.3 *
Chlorophyll	257.9 ***	140.8 ***	4.4 *	3.5 ns	150.3 ***	1.1 ns	1.1 ns	1.5 ns	0.2 ns	0.8 ns	3.0 ns
Shoot Cr conc.	0.2 ns	0.9 ns	3.0 ns	1.4 ns	0.0 ns	0.0 ns	0.1 ns	0.0 ns	0.1 ns	0.0 ns	0.0 ns
Root Cr conc.	0.1 ns	4.7 *	1.6 ns	14.8 ***	0.1 ns	0.2 ns	1.2 ns	0.5 ns	0.0 ns	0.6 ns	0.5 ns
Shoot Fe conc.	1.6 ns	5.6 *	0.1 ns	3.2 ns	0.7 ns	0.1 ns	0.7 ns	0.1 ns	0.2 ns	0.0 ns	0.3 ns
Root Fe conc.	3.4 ns	8.8 **	3.8 ns	0.6 ns	2.5 ns	0.2 ns	0.6 ns	0.5 ns	0.0 ns	0.3 ns	0.0 ns

Significant levels: * *p* < 0.05, ** *p* < 0.01, *** *p* < 0.001; ns: nonsignificant effect.

## Supplementary Materials

The following are available online at https://www.mdpi.com/2305-6304/8/2/26/s1, Figure S1: Characterization of S-nZVI: (a) SEM image; (b) TEM image; (c) XRD pattern, Figure S2: Characterization of B-nZVI: (a) SEM image; (b) XRD pattern, Figure S3: SEM images (upper) and FTIR spectra (lower) of BC, Figure S4: TEM (A-C) and EDS (a-c) images of root samples. (A,a) control roots; (B,b) roots exposed to 100 mg/kg S-nZVI; (C,c) roots exposed to 1000 mg/kg S-nZVI.
