# Supplementary material for: Remediation of Cr(VI)-Contaminated Soil by Nano-Zero-Valent Iron in Combination with Biochar or Humic Acid and the Consequences for Plant Performance
Source: Toxics. 2020 Apr 3;8(2):26. doi: 10.3390/toxics8020026 (PMC7357137; doi:10.3390/toxics8020026)
Supplement: Supplementary file 1 [file toxics-08-00026-s001.pdf]

Supplementary Materials: Remediation of Cr(VI)-Contaminated Soil by Nano-Zero-Valent Iron in Combination with Biochar or Humic Acid and the Consequences for Plant Performance

Yuhuan Sun, Fangyuan Zheng, Wenjie Wang, Shuwu Zhang and Fayuan Wang

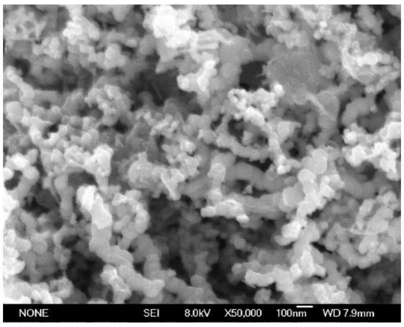

(**a**)

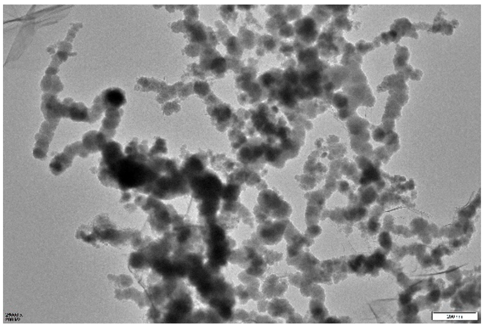

(**b**)

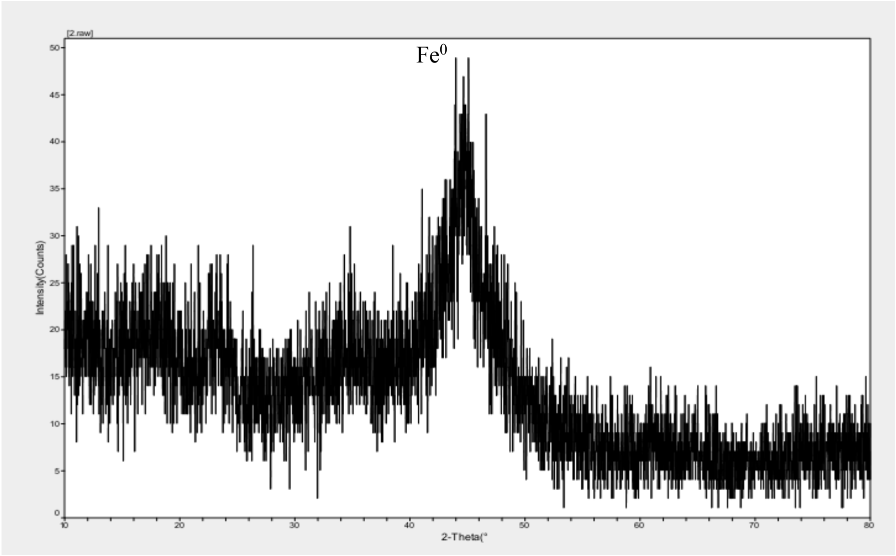

(**c**)

Figure S1. Characterization of S-nZVI: (**a**) SEM image; (**b**) TEM image; (**c**) XRD pattern.

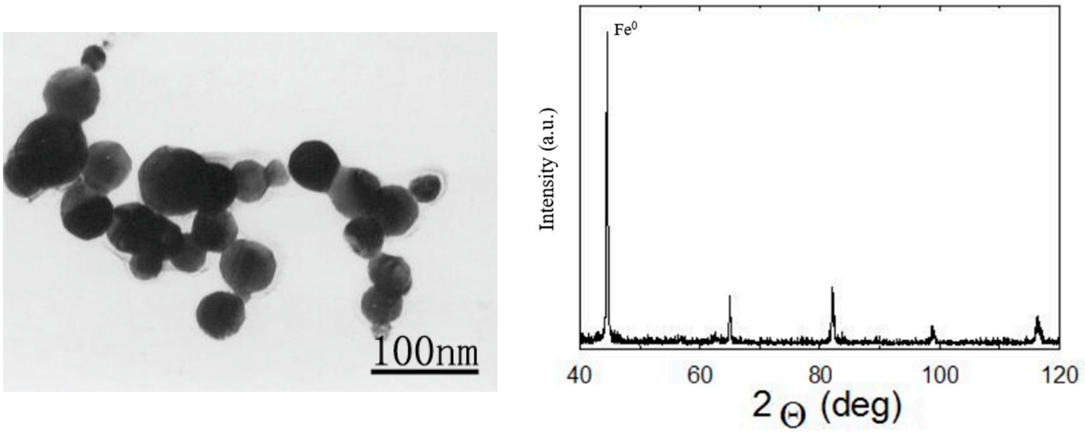

(**a**) (**b**)

Figure S2. Characterization of B-nZVI: (**a**) SEM image; (**b**) XRD pattern.

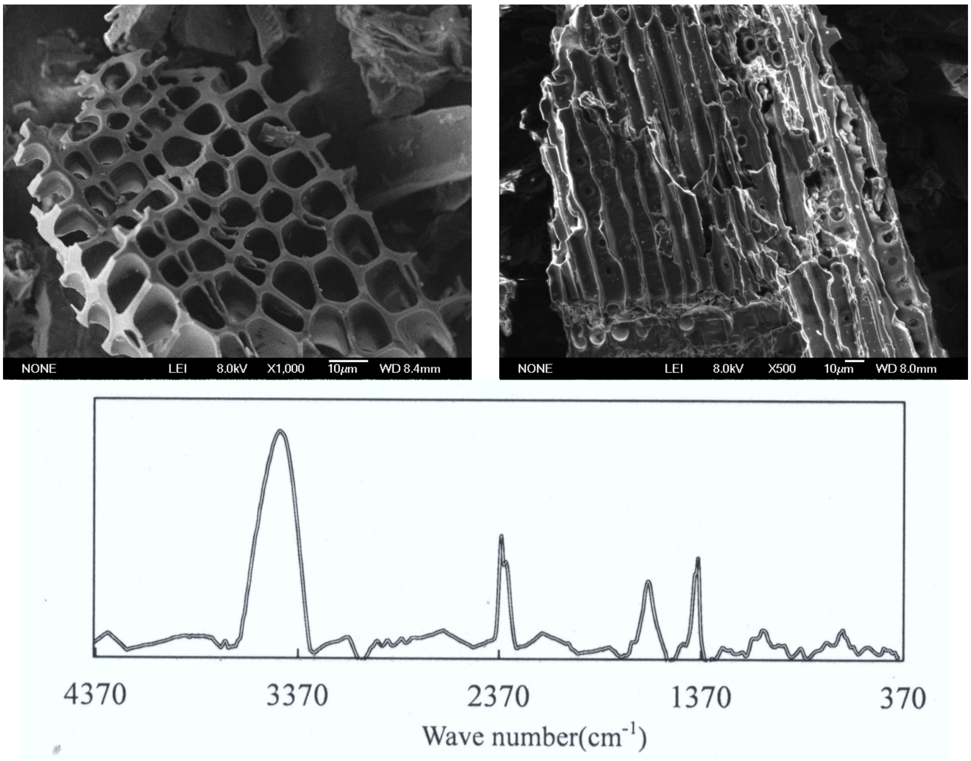

Figure S3. SEM images (upper) and FTIR spectra (lower) of biochar.

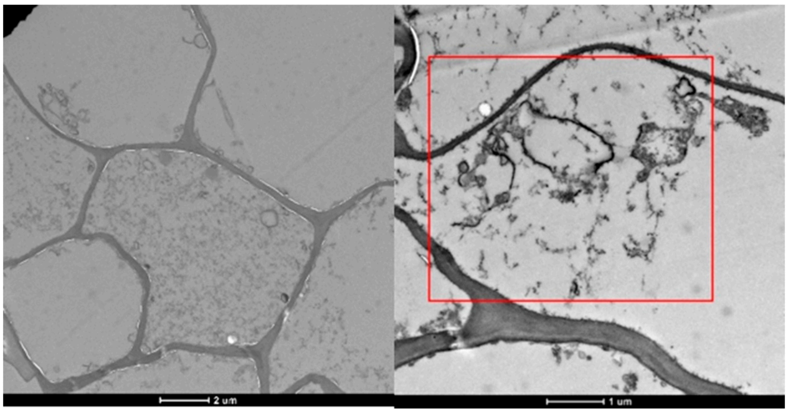
2 um
1 um

(**A**)

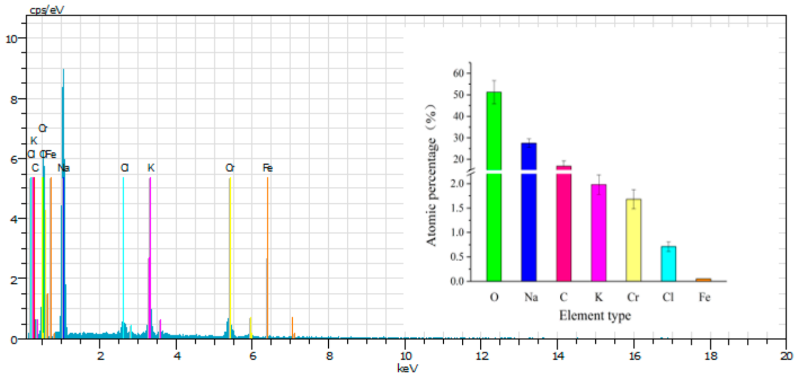
cps/eV
keV
C
K
Cl
Cr
Fe
Na
Cl
K
Cr
Fe
Atomic percentage（%）
Element type
O
Na
C
K
Cr
Cl
Fe

(a)

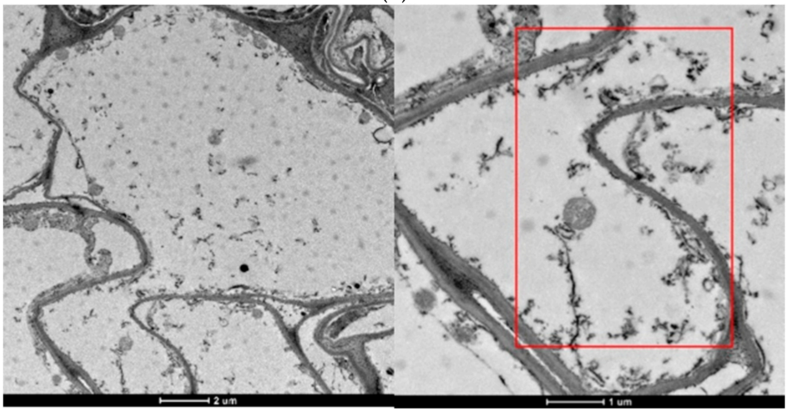
2 um
1 um

(B)

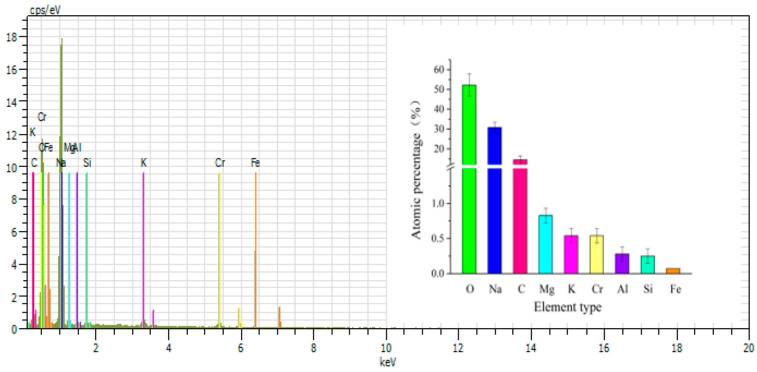
cps/eV
keV
C
K
Cr
Fe
Na
Mg
Al
Si
K
Cr
Fe
Atomic percentage（%）
Element type
O
Na
C
Mg
K
Cr
Al
Si
Fe

(b)

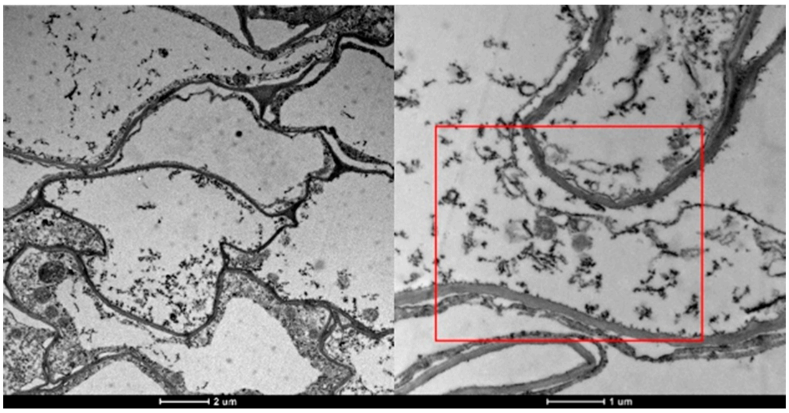

(C)

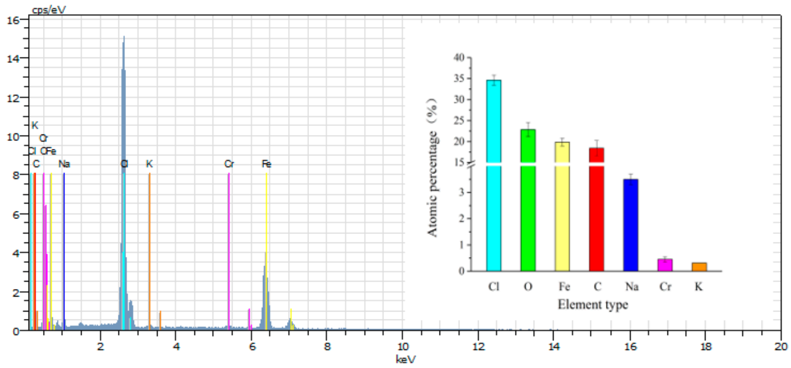

(c)

Figure S4. TEM (**A–C**) and EDS (**a–c**) images of root samples. (A,a) control roots; (B,b) roots exposed to 100 mg/kg S-nZVI; (C,c) roots exposed to 1000 mg/kg S-nZVI.
